# The willingness for dietary and behavioral changes in frontline epidemic prevention workers after experiencing the outbreak of COVID-19 in China: a cross-sectional study

**DOI:** 10.1186/s12199-021-00979-5

**Published:** 2021-05-18

**Authors:** Weijun Yu, Ying Xu, Jianhua Zhang, Qing Yuan, Yanfang Guo, Zhixue Li, Xiangyang He, Yan Ma, Fengmin Cai, Zheng Liu, Rencheng Zhao, Dewang Wang, Jialong Chen, Quanwei Guo

**Affiliations:** 1 Bao’an District Hospital for Chronic Diseases Prevention and Cure, Shenzhen, 518100 China; 2 Shenzhen Hospital, Southern Medical University, Shenzhen, 518101 China; 3 School of Public Health, Guangdong Medical University, Dongguan, 523808 China

**Keywords:** COVID-19, Frontline epidemic prevention workers, Dietary habits, Behavioral habits, Willingness to change, Cross-sectional study

## Abstract

**Background:**

The 2019 novel coronavirus disease (COVID-19) has had a massive impact on public health, resulting in sudden dietary and behavioral habit changes. Frontline epidemic prevention workers play a pivotal role against COVID-19. They must face high-risk infection conditions, insufficient anti-epidemic material supplies, mental pressure, and so on. COVID-19 seriously affects their dietary and behavioral habits, and poor habits make them more susceptible to COVID-19. However, their baseline dietary and behavioral habits before COVID-19 and their willingness to change these habits after the outbreak of COVID-19 remain unclear for these workers in China. This study aimed to explore the baseline dietary and behavioral habits of frontline workers and their willingness to change these habits after the outbreak of the epidemic; in addition, susceptible subgroups were identified by stratified analyses as targets of protective measures to keep them from being infected with COVID-19.

**Methods:**

A cross-sectional study was conducted through an online questionnaire using a sample of 22,459 valid individuals living in China, including 9402 frontline epidemic prevention workers.

**Results:**

Before COVID-19, 23.9% of the frontline epidemic prevention workers reported a high-salt diet, 46.9% of them reported a high frequency of fried foods intake, and 50.9% of them smoked cigarettes. After the outbreak of COVID-19, 34.6% of them expressed a willingness to reduce salt intake, and 43.7% of them wanted to reduce the frequency of pickled vegetables intake. A total of 37.9% of them expressed a willingness to decrease or quit smoking, and 44.5% of them wanted to increase sleep duration. Significant differences in the baseline dietary and behavioral habits and the willingness to change their habits were observed between frontline epidemic prevention workers and other participants.

Among the frontline epidemic prevention workers with poor dietary and behavioral habits before COVID-19, frontline epidemic prevention experience was a promoting factor for adopting worse dietary and behavioral habits, including those in the high-salt intake subgroup (OR, 2.824; 95% CI, 2.341–3.405) and the 11–20 cigarettes/day subgroup (OR, 2.067; 95% CI, 1.359–3.143).

**Conclusions:**

The dietary and behavioral habits of frontline epidemic prevention workers were worse than that those of other participants before COVID-19. They had a greater willingness to adopt healthy dietary and behavioral habits after experiencing the outbreak of COVID-19. However, frontline epidemic prevention workers with poor dietary and behavioral habits before COVID-19 continued in engage in these poor habits. Dietary and behavioral intervention policies should be drafted to protect their health, especially frontline epidemic prevention workers with poor habits at baseline.

## Background

On January 30, 2020, the World Health Organization (WHO) Emergency Committee declared the 2019 novel coronavirus disease (COVID-19) a global health emergency [1]. It quickly spread worldwide and has become a public health emergency. As of December 31, 2020, the death toll of COVID-19 exceeded 1.7 million and the number of confirmed cases was more than 81 million worldwide [2]. COVID-19 has caused great harm to human health and the economy [3, 4], which has aroused widespread concern in society [5, 6]. Recent data suggested that only a few countries were able to prevent the spread of the epidemic and most countries are facing great challenges with COVID-19, especially in South Asia, South and North America, and Europe [7]. Moreover, some countries have suffered a second wave, and there may be more waves of coronavirus infection with the mutated coronavirus strains, with premature relaxation of interventions, with the economy restarting and with cool weather in the Northern Hemisphere [8, 9].

In this tough global fight against COVID-19, frontline epidemic prevention workers play a crucial role [10]. However, it has been reported that many frontline epidemic prevention workers have been infected and even died [11, 12]. They must face large pressures with heavy workloads, high risk of infection, mental pressure, and depression and anxiety during the COVID-19 period [13–18]. Given the great influence of COVID-19, everyone had to change their dietary and behavioral habits to adapt to this current serious situation, including frontline epidemic prevention workers [19]. Willett et al. reported that unhealthy behaviors and lifestyles would lead to people suffering from low immunity and chronic diseases [20]. Studies have revealed that immunocompromised people and patients with chronic diseases have higher COVID-19 infection rates and mortality after infection [21–24]. However, the baseline dietary and behavioral habits of frontline epidemic prevention workers before COVID-19 and their willingness to change these habits after the outbreak of COVID-19 remain unclear in China. Therefore, research data are needed to develop evidence-driven strategies to keep frontline epidemic prevention workers healthy and to reduce their risk of being infected during the epidemic.

In this study, we investigated the baseline dietary and behavioral habits of frontline epidemic prevention workers before COVID-19 and further explored the relationship between the frontline epidemic prevention experience during the epidemic outbreak and their willingness to change dietary and behavioral habits after the outbreak of COVID-19 based on a cross-sectional study in China. To further explore targeted strategies, those who expressed a willingness to adopt worse dietary and behavioral habits were screened out by a stratified analysis. Our work will be useful for governments or organizations to develop interventions to protect frontline epidemic prevention workers from COVID-19, especially in subgroups with poor dietary and behavioral habits.

## Methods

### Participants

After the outbreak of COVID-19 in China, a cross-sectional study was conducted online for all netizens on April 25, 2020. The participants were recruited by the snowball sampling method, and this nonprobability method had the advantages of convenience, high efficiency, and avoidance of direct contact during COVID-19 [25]. The inclusion criteria were as follows: (1) they all volunteered to participate in this questionnaire survey, and (2) they independently completed the questionnaire without interference or logical errors. We defined frontline epidemic prevention workers as medical workers, community workers, policemen, volunteers, and others (anti-epidemic officers especially grassroots cadres, taxi drivers, delivery men, cleaners, construction workers of epidemic prevention facilities, workers who transfer people diagnosed or suspected of being infected with COVID-19, etc.). A total of 28,877 responders participated in the study in China.

### Data collection

The online anonymous questionnaire, which permitted more authentic and reliable data than traditional paper questionnaires, was published in the WeChat public account (such as Twitter) of Bao’an District Hospital for Chronic Diseases Prevention and Cure. All participants could complete the online questionnaires by scanning a quick response code. Unified guidance language was used to introduce the study purposes and ensured data confidentiality to the participants. The system could intelligently prompt participants until the questionnaire was completed and submitted. Of the 28,877 responders, 1966 dropped out, 4452 were excluded for logical errors, and 22,459 were effective. A total of 9402 frontline epidemic prevention workers’ valid questionnaires were collected, accounting for 41.9%.

### Questionnaire

A structured questionnaire (Table 1) with close-ended questions was developed after searching the literature [26] and consulting experts and included the following: (1) General characteristics: gender, age, height, weight, education level, marital status, occupation, main living place, and frontline epidemic prevention experience during the outbreak of COVID-19. (2) Baseline dietary habits before COVID-19: salt intake (high, medium, and low), intake frequency (days/week) of fried foods, sugary foods, pickled vegetables, processed meat products, fresh vegetables, fresh fruits, and protein-rich products. We defined sugary foods as sugar, sugary beverages, desserts, cookies, candies, fruit products, and dairy desserts and protein-rich products as meat, eggs, milk, and dairy. (3) The baseline behavioral habits before COVID-19: smoking (cigarettes/day), alcohol consumption frequency (days/week), sleep duration (hours/day), and physical exercise time (minutes or hours/week). (4) Willingness to change dietary habits after experiencing the outbreak of COVID-19: self-reported willingness to change (unchanged, increase, decrease, uncertain) dietary habits, including intake of salt, fried foods, sugary foods, pickled vegetables, processed meat products, fresh vegetables, fresh fruits, and protein-rich products after experiencing the outbreak of COVID-19. (5) Willingness to change behavioral habits after experiencing the outbreak of COVID-19: self-reported willingness to change behavioral habits, including smoking, alcohol consumption frequency, sleep duration, and physical exercise time after experiencing the outbreak of COVID-19.

**Table 1:** Investigated questions on baseline dietary and behavioral habits, and willingness to change dietary and behavioral habits after experiencing the outbreak of COVID-19

Investigated questions	
**Baseline dietary habits**	
Salt intake: “Before COVID-19, What about your dietary intake of salt?”	
Intake frequency of fresh vegetables: “Before COVID-19, how often did you normally eat fresh vegetables?”	
Intake frequency of fresh fruits: “Before COVID-19, how often did you normally eat fresh fruits?”	
Intake frequency of meat, eggs, milk, or dairy products: “Before COVID-19, how often did you normally eat meat, eggs, milk, or dairy products?”	
Intake frequency of fried foods: “Before COVID-19, how often did you normally eat fried foods?”	
Intake frequency of sugary foods: “Before COVID-19, how often did you normally eat sugar or sugary foods (including sugary drinks, desserts, biscuits, sweets, fruit products, dairy desserts, etc.)?”	
Intake frequency of pickled vegetables: “Before COVID-19, how often did you normally eat pickled vegetables?”	
Intake frequency of processed meat: “Before COVID-19, how often did you normally eat processed meat?”	
**Baseline behavioral habits**	
Smoking cigarettes per day: “Before COVID-19, how many cigarettes did you normally smoke per day?”	
Frequency of alcohol consumption: “Before COVID-19, how often did you normally drink alcohol?”	
Sleep duration (including lunch break): “Before COVID-19, how long often did you sleep (including lunch break)?”	
Physical exercise time per week: “Before COVID-19, how long often did your physical exercise per week?”	
**Willingness to change dietary habits**	
Salt: “Do you plan or are you changing salt intake after experiencing the outbreak of COVID-19?”	
Fresh vegetables: “Do you plan or are you changing the intake frequency of fresh vegetables after experiencing the outbreak of COVID-19?”	
Fresh fruits: “Do you plan or are you changing the intake frequency of fresh fruits after experiencing the outbreak of COVID-19?”	
Meat, eggs, milk, or dairy products: “Do you plan or are you changing the intake frequency of meat, eggs, milk, or dairy products after experiencing the outbreak of COVID-19?”	
Fried foods: “Do you plan or are you changing the intake frequency of fried foods after experiencing the outbreak of COVID-19?”	
Sugary foods: “Do you plan or are you changing the intake frequency of sugary foods after experiencing the outbreak of COVID-19?”	
Pickled vegetables: “Do you plan or are you changing the intake frequency of pickled vegetables after experiencing the outbreak of COVID-19?”	
Processed meat: “Do you plan or are you changing the intake frequency of processed meat after experiencing the outbreak of COVID-19?”	
**Willingness to change behavioral habits**	
Smoking: “Do you plan or are you changing the number of smoking cigarettes per day after experiencing the outbreak of COVID-19?”	
Alcohol consumption: “Do you plan or are you changing the frequency of alcohol consumption after experiencing the outbreak of COVID-19?”	
Sleep: “Do you plan or are you changing sleep duration after experiencing the outbreak of COVID-19?”	
Physical exercise: “Do you plan or are you changing physical exercise time after experiencing the outbreak of COVID-19?”	

### Statistical methods

The original data were downloaded directly from the website. The SPSS 25.0 software package was used for statistical analysis. Measurement data (age and BMI) were analyzed by Student’s *t*-tests. Categorical data were analyzed with chi-square tests. Salt intake (low = 1, medium = 2, and high = 3), intake frequency (≤ 1 day/week = 1, 2–3 days/week = 2, 4–6 days/week = 3, every day = 4), smoking (no smoking = 1, 1–5 cigarettes/day = 2, 6–10 cigarettes/day = 3, 11–20 cigarettes/day = 4, > 20 cigarettes/day = 5), sleep duration (< 5 h/day = 1, 5–6 h/day = 2, 6–7 h/day = 3, 7–8 h/day = 4, > 8 h/day = 5), and physical exercise (< 30 min/week = 1, 30–60 min/week = 2, 1–2 h/week = 3, 2–3 h/week = 4, > 3 h/week = 5) were treated as ordinal variables. Gender, frontline epidemic prevention experience, and current residence place were treated as dichotomous variables. Education level, marital status, occupation, and willingness to change dietary and behavioral habits were regarded as nominal variables. The baseline dietary and behavioral habits were regarded as a stratification factor and those with unchanged willingness were regarded as a reference group in the multinomial logistic regression models. After adjusting for other characteristic factors (gender, age, BMI, education level, marital status, occupation, and current residence place), the willingness to change dietary and behavioral habits were investigated in frontline epidemic prevention workers after experiencing the outbreak of COVID-19. For all analyses, the difference was statistically significant at *P* < 0. 05.

## Results

### General information from the questionnaires

From 10:00 on January 23, 2020 to 0:00 on April 8, 2020, Wuhan engaged in anti-epidemic warfare for these 76 days and then officially restarted; we defined this period as the outbreak of COVID-19 in China. To investigate frontline epidemic prevention workers’ baseline dietary and behavioral habits before COVID-19, and their willingness to change dietary and behavioral habits after experiencing the outbreak of COVID-19, online questionnaires were conducted on April 25, 2020.

A total of 22,459 responders participated in this study, including 9402 (41.9%) frontline epidemic prevention workers, and 13,057 (58.1%) non-frontline epidemic prevention workers. Among them, 14,204 were males (63.2%), and 8255 were females (36.8%); 11,182 (49.8%) were married, 10,567 (47.1%) were unmarried, and 710 (3.2%) were others (including remarried, cohabiting, separated, divorced, and widowed); and 20,650 (91.9%) were in senior high school and above, and 1809 (8.1%) were in junior high school and below. The mean age and BMI were 27.88 ± 7.84 (years) and 22.05 ± 4.88, respectively. During the outbreak of COVID-19, 14,069 (62.6%) lived in Guangdong Province, 292 (1.3%) lived in Hubei, and 8098 (36.1%) lived in other provinces (Table 2).

**Table 2:** Demographic characteristics of participants according to frontline epidemic prevention experience during the outbreak of COVID-19

Demographic characteristics	Frontline epidemic prevention experience, ***N*** (%)		Total, ***N*** = 22,459	***T*** or χ^**2**^	***P-***value
	Yes, 9402 (41.9)	No, 13,057 (58.1)			
Gender					
Male	6392 (68.0)	7812 (59.8)	14,204 (63.2)	156.399	< 0.001
Female	3010 (32.0)	5245 (40.2)	8255 (36.8)		
Age (years), mean ± SD	28.09 ± 7.50	27.73 ± 8.08	27.88 ± 7.84	− 3.457	0.001
BMI, mean ± SD	21.93 ± 4.85	22.13 ± 4.91	22.049 ± 4.88	3.058	0.002
Marital status					
Married	5035 (53.6)	6147 (47.1)	11,182 (49.8)	121.955	< 0.001
Never-married	4022 (42.8)	6545 (50.1)	10,567 (47.1)		
Others^a^	345 (3.7)	365 (2.8)	710 (3.2)		
Education					
Primary schools and below	172 (1.8)	164 (1.3)	336 (1.5)	38.409	< 0.001
Middle school	602 (6.4)	871 (6.7)	1473 (6.6)		
High school or technical secondary school	2292 (24.4)	3582 (27.4)	5874 (26.2)		
College	2790 (29.7)	3717 (28.5)	6507 (29.0)		
Bachelor’s degree and above	3546 (37.7)	4723 (36.2)	8269 (36.8)		
Occupation					
Medical workers	478 (5.1)	233 (1.8)	711 (3.2)	421.452	< 0.001
Community worker	620 (6.6)	391 (3.0)	1011 (4.5)		
Policemen	124 (1.2)	68 (0.5)	192 (0.9)		
Other	8180 (87.0)	12,365 (94.7)	20,545 (91.5)		
Place of current residence					
Hubei	121 (1.3)	171 (1.3)	292 (1.3)	0.022	0.882
Other parts of China	9281 (98.7)	12,886 (98.7)	22,167 (98.7)		

^a^Others included remarried, cohabiting, separated, divorced, and widowed

### Information of the frontline epidemic prevention workers

A total of 9402 frontline epidemic prevention workers’ valid questionnaires were collected. Among them, 6392 were males (68.0%) and 3010 were females (32.0%); 5035 (53.6%) were married, 4022 (42.8%) were unmarried, and 345 (3.7%) were others; 6336 (67.4%) were in college and above and 3066 (32.6%) in senior high school and below; 478 (5.1%) were medical workers, 620 (6.6%) were community workers, 124 (1.3%) were policemen, and 8180 (87.0%) were others. The mean age and BMI were 28.09 ± 7.50 (years) and 21.93 ± 4.85, respectively. During the outbreak of COVID-19, 121 (1.3%) lived in Hubei Province, and 9821 (98.7%) lived in other provinces (Table 2).

### Univariate analysis of associations between frontline epidemic prevention experience and baseline dietary and behavioral habits before COVID-19

Before COVID-19, 23.9% of the frontline epidemic prevention workers reported that they had a high-salt diet, and 46.9%, 55.1%, 45.0%, and 49.1% of them reported that their intake frequency of fried foods, sugary foods, pickled vegetables, and processed meat products, respectively, was 4–6 days/week or above. However, 21.6%, 26.8%, and 30.5% of them reported that their intake frequency of fresh vegetables, fresh fruits, and protein-rich products, respectively, was 2–3 days/week or below. Most of these data for the frontline epidemic prevention workers were higher than the data for the other participants, and these differences in dietary habits were statistically significant (*P* < 0.05) (Table 3).

**Table 3:** Univariate analysis of associations between frontline epidemic prevention experience and baseline dietary and behavioral habits before COVID-19

Baseline characteristics	Frontline epidemic prevention experience, ***N*** (%)		Total, ***N*** = 22,459	***T*** or ***χ***^***2***^	***P***-value
	Yes, 9402 (41.9)	No, 13,057 (58.1)			
**Baseline dietary habits**					
Salt intake					
High	2244 (23.9)	2610 (20.0)	4854 (21.6)	50.693	< 0.001
Medium	5767 (61.3)	8322 (63.7)	14,089 (62.7)		
Low	1391 (14.8)	2125 (16.3)	3516 (15.7)		
Intake frequency of fresh vegetables					
Every day	3987 (42.4)	6561 (50.2)	10,548 (47.0)	153.388	< 0.001
4–6 days/week	3382 (36.0)	3810 (29.2)	7192 (32.0)		
2–3 days/week	1791 (19.0)	2355 (18.0)	4146 (18.5)		
≤ 1 day/week	242 (2.6)	331 (2.5)	573 (2.6)		
Intake frequency of fresh fruits					
Every day	3510 (37.3)	5233 (40.1)	8743 (38.9)	107.917	< 0.001
4–6 days/week	3379 (35.9)	3903 (29.9)	7282 (32.4)		
2–3 days/week	2177 (23.2)	3255 (24.9)	5432 (24.2)		
≤ 1 day/week	336 (3.6)	666 (5.1)	1002 (4.5)		
Intake frequency of meat, eggs, milk, or dairy products					
Every day	3113 (33.1)	4779 (36.6)	7892 (35.1)	87.739	< 0.001
4–6 days/week	3430 (36.5)	4041 (30.9)	7471 (33.3)		
2–3 days/week	2459 (26.2)	3518 (26.9)	5977 (26.6)		
≤ 1 day/week	400 (4.3)	719 (5.5)	1119 (5.0)		
Intake frequency of fried foods					
Every day	1417 (15.1)	1574 (12.1)	2991 (13.3)	372.657	< 0.001
4–6 days/week	2991 (31.8)	3183 (24.4)	6174 (27.5)		
2–3 days/week	2995 (31.9)	4135 (31.7)	7130 (31.7)		
≤ 1 day/week	1999 (21.3)	4165 (31.9)	6164 (27.4)		
Intake frequency of sugary foods					
Every day	2032 (21.6)	2643 (20.2)	4675 (20.8)	195.740	< 0.001
4–6 days/week	3150 (33.5)	3569 (27.3)	6719 (29.9)		
2–3 days/week	3144 (33.4)	4625 (35.4)	7769 (34.6)		
≤ 1 day/week	1076 (11.4)	2220 (17.0)	3296 (14.7)		
Intake frequency of pickled vegetables					
Every day	1426 (15.2)	1726 (13.2)	3152 (14.0)	419.729	< 0.001
4–6 days/week	2801 (29.8)	2972 (22.8)	5773 (25.7)		
2–3 days/week	3179 (33.8)	4007 (30.7)	7186 (32.0)		
≤ 1 day/week	1996 (21.2)	4352 (33.3)	6348 (28.3)		
Intake frequency of processed meat products					
Every day	1548 (16.5)	1848 (14.2)	3396 (15.1)	380.866	< 0.001
4–6 days/week	3065 (32.6)	3240 (24.8)	6305 (28.1)		
2–3 days/week	3210 (34.1)	4465 (34.2)	7675 (34.2)		
≤ 1 day/week	1579 (16.8)	3504 (26.8)	5083 (22.6)		
**Baseline behavioral habits**					
Smoking cigarettes/day					
> 20 cigarettes per day	148 (1.6)	135 (1.0)	283 (1.3)	721.002	< 0.001
11–20 cigarettes per day	694 (7.4)	727 (5.6)	1421 (6.3)		
6–10 cigarettes per day	1971 (21.0)	1732 (13.3)	3703 (16.5)		
1–5 cigarettes per day	1963 (20.9)	1726 (13.2)	3689 (16.4)		
No smoking	4626 (49.2)	8737 (66.9)	13,363 (59.5)		
Alcohol consumption/week					
Every day	992 (10.6)	1017 (7.8)	2009 (8.9)	1024.661	< 0.001
4–6 days/week	1813 (19.3)	1386 (10.6)	3199 (14.2)		
2–3 days/week	2309 (24.6)	2214 (17.0)	4523 (20.1)		
≤ 1 day/week	1537 (16.3)	2021 (15.5)	3558 (15.8)		
No drinking	2751 (29.3)	6419 (49.2)	9170 (40.8)		
Sleep duration/day (including lunch break)					
< 5 h/day	800 (8.5)	777 (6.0)	1577 (7.0)	231.381	< 0.001
5–6 h/day	1799 (19.1)	1867 (14.3)	3666 (16.3)		
6–7 h/day	3000 (31.9)	3986 (30.5)	6986 (31.1)		
7–8 h/day	2851 (30.3)	4668 (35.8)	7519 (33.5)		
> 8 h/day	952 (10.1)	1759 (13.5)	2711 (12.1)		
Physical exercise time/week					
< 30 min/week	1682 (17.9)	3772 (28.9)	5454 (24.3)	424.613	< 0.001
30 min–1 h/week	3401 (36.2)	4614 (35.3)	8015 (35.7)		
1–2 h/week	2752 (29.3)	2907 (22.3)	5659 (25.2)		
2–3 h/week	1081 (11.5)	1136 (8.7)	2217 (9.9)		
> 3 h/week	486 (5.2)	628 (4.8)	1114 (5.0)		

Of the frontline epidemic prevention workers, 50.9% of them self-reported that they smoked cigarettes, 29.9% of them reported that their alcohol consumption frequency was 4–6 days/week or above, 59.5% of them reported that their sleep duration was less than 7 h/day, and 54.1% of them reported that their physical exercise time was less than 1 h/week before COVID-19. Most of these data for the frontline epidemic prevention workers were higher than the data for other participants, and these differences in behavioral habits were statistically significant (*P* < 0.05) (Table 3).

### Univariate analysis of associations between frontline epidemic prevention experience and willingness to change dietary and behavioral habits after experiencing the outbreak of COVID-19

After experiencing the outbreak of COVID-19, 34.6% of the frontline epidemic prevention workers reported their willingness to reduce their salt intake, and 45.2%, 40.7%, 43.7%, and 40.5% of them reported their willingness to reduce the frequency of their intake of fried foods, sugary foods, pickled vegetables, and processed meat products, respectively. Meanwhile, 52.1%, 50.0%, and 43.2% of them reported their willingness to increase the frequency of their intake of fresh vegetables, fresh fruits, and protein-rich products, respectively. Most of these data for the frontline epidemic prevention workers were higher than that the data for the other participants, and significant differences in willingness to change dietary habits were observed (*P* < 0.05) (Table 4).

**Table 4:** Univariate analysis of associations between frontline epidemic prevention experience and willingness to change dietary and behavioral habits after experiencing the outbreak of COVID-19

Variables characteristics	Frontline epidemic prevention experience, ***N*** (%)		Total, ***n*** = 22,459	***T*** or ***χ***^***2***^	***P-***value
	Yes, 9402 (41.9)	No, 13,057 (58.1)			
**Willingness to change dietary habits**					
Salt intake					
Unchange	3741 (39.8)	6683 (51.2)	10,424 (46.4)	582.069	< 0.001
Increase	2000 (21.3)	1536 (11.8)	3536 (15.7)		
Decrease	3257 (34.6)	3900 (29.9)	7157 (31.9)		
Uncertain	404 (4.3)	938 (7.2)	1342 (6.0)		
Intake frequency of fresh vegetables					
Unchange	2892 (29.8)	5202 (39.8)	8004 (35.6)	310.811	< 0.001
Increase	4896 (52.1)	5774 (44.2)	10,670 (47.5)		
Decrease	1399 (14.9)	1467 (11.2)	2866 (12.8)		
Uncertain	305 (3.2)	614 (4.7)	919 (4.1)		
Intake frequency of fresh fruits					
Unchange	2811 (29.9)	5059 (38.7)	7870 (35.0)	304.083	< 0.001
Increase	4698 (50.0)	5786 (44.3)	10,484 (46.7)		
Decrease	1594 (11.9)	1553 (11.9)	3147 (14.0)		
Uncertain	299 (3.2)	659 (5.0)	958 (4.3)		
Intake frequency of meat, eggs, milk, or dairy products					
Unchange	3059 (32.5)	5697 (43.6)	8756 (39.0)	365.679	< 0.001
Increase	4063 (43.2)	4499 (34.5)	8562 (38.1)		
Decrease	1920 (20.4)	2137 (16.4)	4057 (18.1)		
Uncertain	360 (3.8)	724 (5.5)	1084 (4.8)		
Intake frequency of fried foods					
Unchange	2344 (24.9)	4253 (32.6)	6597 (29.4)	486.665	< 0.001
Increase	2437 (25.9)	1957 (15.0)	4394 (19.6)		
Decrease	4254 (45.2)	6080 (46.6)	10,334 (46.0)		
Uncertain	367 (3.9)	767 (5.9)	1134 (5.0)		
Intake frequency of sugary foods					
Unchange	2523 (26.8)	4654 (35.6)	7177 (32.0)	552.086	< 0.001
Increase	2617 (27.8)	2091 (16.0)	4708 (21.0)		
Decrease	3822 (40.7)	5378 (41.2)	9200 (41.0)		
Uncertain	440 (4.7)	934 (7.2)	1374 (6.1)		
Intake frequency of pickled vegetables					
Unchange	2375 (25.3)	4411 (33.8)	6786 (30.2)	462.560	< 0.001
Increase	2437 (25.9)	2062 (15.8)	4499 (20.0)		
Decrease	4113 (43.7)	5602 (42.9)	9715 (43.3)		
Uncertain	477 (5.1)	982 (7.5)	1459 (6.5)		
Intake frequency of processed meat products					
Unchange	2548 (27.1)	4495 (34.4)	7043 (31.4)	413.992	< 0.001
Increase	2573 (27.4)	2246 (17.2)	4819 (21.5)		
Decrease	3809 (40.5)	5319 (40.7)	9128 (40.6)		
Uncertain	472 (5.0)	997 (7.6)	1469 (6.5)		
**Willingness to change behavioral habits**					
Smoking cigarettes/day					
Unchange	4074 (43.3)	7867 (60.3)	11,941 (53.2)	774.274	< 0.001
Increase	1565 (16.6)	1111 (8.5)	2676 (11.9)		
Decrease	2400 (25.5)	2362 (18.1)	4762 (21.2)		
Quit smoking	1163 (12.4)	1341 (10.3)	2504 (11.1)		
Uncertain	200 (2.1)	376 (2.9)	576 (2.6)		
Alcohol consumption/week					
Unchange	3570 (38.0)	6998 (53.6)	10,568 (47.1)	720.118	< 0.001
Increase	1552 (16.5)	1173 (9.0)	2725 (12.1)		
Decrease	2815 (29.9)	2891 (22.1)	5706 (25.4)		
Quit drinking	1184 (12.6)	1401 (10.7)	2585 (11.5)		
Uncertain	281 (3.0)	594 (4.5)	875 (3.9)		
Sleep duration/day					
Unchange	2793 (29.7)	5272 (40.4)	8065 (35.9)	349.024	< 0.001
Increase	4181 (44.5)	5029 (38.5)	9210 (41.0)		
Decrease	1964 (20.9)	1945 (14.9)	3909 (17.4)		
Uncertain	464 (4.9)	811 (6.2)	1275 (5.7)		
Physical exercise time/week					
Unchange	2236 (23.8)	3765 (28.8)	6001 (26.7)	173.142	< 0.001
Increase	5387 (57.3)	7284 (55.8)	12,671 (56.4)		
Decrease	1516 (16.1)	1479 (11.3)	2995 (13.3)		
Uncertain	263 (2.8)	529 (4.1)	792 (3.5)		

A total of 37.9% and 42.5% of the frontline epidemic prevention workers reported their willingness to decrease or quit their smoking and drinking alcohol consumption, and 44.5% and 53.7% of them had a willingness to increase sleep duration and physical exercise time, respectively. All these data for the frontline epidemic prevention workers were higher than the data for other participants, and significant differences in willingness to change behavioral habits between them were observed (*P* < 0.05) (Table 4).

### Multivariable analysis of associations between frontline epidemic prevention experience and willingness to change dietary habits

With baseline dietary habit as a stratification factor and unwillingness to change dietary habit as a reference group, and after adjustments were made for gender, age, BMI, education level, marital status, occupation, and main living place, the stratified analysis of the multinomial logistic regression models showed that the frontline epidemic prevention workers with bad dietary habits were more likely to choose worse dietary habits after experiencing the outbreak of COVID-19. Regarding salt intake, frontline epidemic prevention workers with high-salt intake were more likely to increase salt intake than other participants (OR, 2.824; 95% CI, 2.341–3.405). Regarding those with the lowest level frequency of fresh fruits (≤ 1 day/week), frontline epidemic prevention workers expressed a willingness to decrease the intake frequency of fresh fruits (OR, 3.000; 95% CI, 1.922–4.682). Moreover, regarding those with the highest-level frequency (every day/week) of sugary foods and fried foods, frontline epidemic prevention workers expressed a willingness to increase their sugary foods intake (OR, 1.683; 95% CI, 1.424–1.988) and fried foods intake (OR, 1.566; 95% CI, 1.271–1.931). Furthermore, the frontline epidemic prevention workers had other kinds of poor dietary habits, including the highest frequency intake of pickled vegetables and processed meat products and the lowest frequency intake of fresh vegetables, and protein-rich products (Table 5).

**Table 5:** Multivariate analysis of associations between frontline epidemic prevention experience and willingness to change dietary habits after experiencing the outbreak of COVID-19

Intake frequency of dietary habits^**a**^	Willingness^**b**^	OR	OR (95% CI)	***P***-value
**Salt intake**				
High	Increase	2.824	2.341–3.405	< 0.001
	Decrease	1.096	0.957–1.254	0.184
	Uncertain	0.512	0.369–0.709	< 0.001
Medium	Increase	2.047	1.859–2.255	< 0.001
	Decrease	1.538	1.418–1.669	< 0.001
	Uncertain	0.800	0.685–0.934	0.005
Low	Increase	1.544	1.210–1.969	0.001
	Decrease	1.737	1.480–2.039	< 0.001
	Uncertain	1.204	0.893–1.622	0.223
Total	Increase	2.114	1.952–2.289	< 0.001
	Decrease	1.448	1.359–1.542	< 0.001
	Uncertain	0.789	0.696–0.895	< 0.001
**Intake frequency of fresh vegetables**				
Every day	Increase	1.400	1.285–1.525	< 0.001
	Decrease	1.407	1.166–1.698	< 0.001
	Uncertain	0.698	0.540–0.901	0.006
4–6 days/week	Increase	1.650	1.456–1.869	< 0.001
	Decrease	1.693	1.442–1.988	< 0.001
	Uncertain	1.104	0.814–1.497	0.525
2–3 days/week	Increase	1.385	1.146–1.673	0.001
	Decrease	1.489	1.207–1.837	< 0.001
	Uncertain	0.975	0.715–1.329	0.872
≤ 1 day/week	Increase	1.661	1.009–2.734	0.046
	Decrease	1.659	0.988–2.787	0.055
	Uncertain	1.041	0.587–1.844	0.891
Total	Increase	1.526	1.436–1.622	< 0.001
	Decrease	1.584	1.450–1.731	< 0.001
	Uncertain	0.907	0.782–1.053	0.199
**Intake frequency of fresh fruits**				
Every day	Increase	1.359	1.237–1.493	< 0.001
	Decrease	1.696	1.384–2.079	< 0.001
	Uncertain	0.693	0.492–0.977	0.036
4–6 days/week	Increase	1.599	1.410–1.813	< 0.001
	Decrease	1.986	1.696–2.327	< 0.001
	Uncertain	1.001	0.750–1.337	0.994
2–3 days/week	Increase	1.495	1.279–1.748	< 0.001
	Decrease	1.806	1.514–2.155	< 0.001
	Uncertain	0.983	0.747–1.295	0.905
≤ 1 day/week	Increase	1.794	1.213–2.653	0.003
	Decrease	3.000	1.922–4.682	< 0.001
	Uncertain	1.343	0.858–2.103	0.197
Total	Increase	1.412	1.328–1.501	< 0.001
	Decrease	1.694	1.555–1.846	< 0.001
	Uncertain	0.820	0.708–0.950	0.008
**Intake frequency of meat, eggs, milk, or dairy products**				
Every day	Increase	1.696	1.521–1.891	< 0.001
	Decrease	1.242	1.038–1.485	0.018
	Uncertain	0.747	0.539–1.036	0.080
4–6 days/week	Increase	1.892	1.682–2.127	< 0.001
	Decrease	1.717	1.491–1.977	< 0.001
	Uncertain	1.011	0.771–1.3273	0.935
2–3 days/week	Increase	1.468	1.277–1.688	< 0.001
	Decrease	1.733	1.490–2.016	< 0.001
	Uncertain	1.042	0.820–1.325	0.737
≤ 1 day/week	Increase	1.252	0.877–1.786	0.216
	Decrease	1.862	1.291–2.685	0.001
	Uncertain	1.232	0.828–1.834	0.303
Total	Increase	1.615	1.518–1.719	< 0.001
	Decrease	1.533	1.437–1.678	< 0.001
	Uncertain	0.923	0.805–1.059	0.254
**Intake frequency of fried foods**				
Every day	Increase	1.566	1.271–1.931	< 0.001
	Decrease	1.152	0.949–1.399	0.153
	Uncertain	0.777	0.460–1.314	0.347
4–6 days/week	Increase	1.769	1.527–2.049	< 0.001
	Decrease	1.191	1.031–1.376	0.018
	Uncertain	1.154	0.834–1.595	0.388
2–3 days/week	Increase	2.635	2.236–3.166	< 0.001
	Decrease	1.486	1.293–1.707	< 0.001
	Uncertain	1.123	0.869–1.451	0.377
≤ 1 day/week	Increase	1.957	1.502–2.548	< 0.001
	Decrease	1.499	1.328–1.692	< 0.001
	Uncertain	1.002	0.806–1.246	0.984
Total	Increase	2.115	1.953–2.290	< 0.001
	Decrease	1.246	1.167–1.330	< 0.001
	Uncertain	0.881	0.768–1.010	0.069
**Intake frequency of sugary foods**				
Every day	Increase	1.683	1.424–1.988	< 0.001
	Decrease	1.049	0.902–1.219	0.534
	Uncertain	0.944	0.663–1.345	0.751
4–6 days/week	Increase	2.273	1.974–2.617	< 0.001
	Decrease	1.446	1.259–1.661	< 0.001
	Uncertain	0.931	0.699–1.239	0.623
2–3 days/week	Increase	2.332	2.009–2.708	< 0.001
	Decrease	1.437	1.276–1.618	< 0.001
	Uncertain	1.160	0.937–1.437	0.172
≤ 1 day/week	Increase	2.562	1.896–3.462	< 0.001
	Decrease	1.553	1.307–1.845	< 0.001
	Uncertain	1.028	0.801–1.319	0.830
Total	Increase	2.134	1.967–2.304	< 0.001
	Decrease	1.269	1.189–1.355	< 0.001
	Uncertain	0.879	0.775–0.996	0.043
**Intake frequency of pickled vegetables**				
Every day	Increase	1.526	1.244–1.873	< 0.001
	Decrease	1.073	0.888–1.297	0.465
	Uncertain	0.946	0.621–1.441	0.795
4–6 days/week	Increase	1.730	1.489–2.010	< 0.001
	Decrease	1.193	1.027–1.386	0.021
	Uncertain	0.940	0.680–1.300	0.709
2–3 days/week	Increase	1.869	1.597–2.188	< 0.001
	Decrease	1.388	1.213–1.587	< 0.001
	Uncertain	1.180	0.929–1.497	0.175
≤ 1 day/week	Increase	2.451	1.901–3.159	< 0.001
	Decrease	1.601	1.416–1.811	< 0.001
	Uncertain	1.072	0.887–1.297	0.470
Total	Increase	2.026	1.873–2.191	< 0.001
	Decrease	1.325	1.241–1.415	< 0.001
	Uncertain	0.906	0.801–1.024	0.114
**Intake frequency of processed meat products**				
Every day	Increase	1.505	1.241–1.826	< 0.001
	Decrease	1.170	0.973–1.407	0.095
	Uncertain	0.759	0.478–1.207	0.244
4–6 days/week	Increase	1.634	1.423–1.876	< 0.001
	Decrease	1.095	0.953–1.259	0.200
	Uncertain	0.914	0.686–1.219	0.542
2–3 days/week	Increase	2.000	1.731–2.311	< 0.001
	Decrease	1.402	1.240–1.584	< 0.001
	Uncertain	1.032	0.825–1.291	0.784
≤ 1 day/week	Increase	1.616	1.233–2.117	0.001
	Decrease	1.577	1.371–1.813	< 0.001
	Uncertain	1.176	0.962–1.439	0.114
Total	Increase	1.885	1.747–2.034	< 0.001
	Decrease	1.234	1.156–1.318	< 0.001
	Uncertain	0.855	0.756–0.956	0.011

^a^Baseline intake frequency of dietary habits was regarded as a stratification factor

^b^Unwillingness to change dietary habits was regarded as a reference group

^c^Frontline epidemic prevention experience was classified into dichotomous variables in the models (regarding “have the frontline epidemic prevention experience” as 1, and “no frontline epidemic prevention experience” as 2), adjusting for other characteristic factors (gender, age, BMI, education level marital status, occupation, and main living place)

### Multivariable analysis of associations between frontline epidemic prevention experience and willingness to change behavioral habits

Using the same method as described in the “Multivariable analysis of associations between frontline epidemic prevention experience and willingness to change dietary habits” section, the data showed that frontline epidemic prevention workers who smoked 1–10 cigarettes/day were not willing to quit smoking but would increase the number of more cigarettes smoked. Regarding those with the highest frequency of alcohol consumption (every day/week), the frontline epidemic prevention experience showed a positive correlation with a willingness to increase the frequency of consuming alcohol (OR, 1.422; 95% CI, 1.085–1.865). The frontline epidemic prevention workers with 5–6 h of sleep duration expressed a willingness to decrease their sleep time (OR, 1.634; 95% CI, 1.301–2.053). The frontline epidemic prevention workers who engaged in physical exercise less than 30 min/week, expressed a willingness to decrease their physical exercise time (OR, 1.379; 95% CI, 1.041–1.828). Our data suggested that some of the frontline epidemic prevention workers expressed a willingness to adopt worse behavioral habits after experiencing the outbreak of COVID-19, especially among those in poor habits subgroups (Table 6).

**Table 6:** Multivariate analysis of associations between frontline epidemic prevention experience and willingness to change behavioral habits after experiencing the outbreak of COVID-19

Intake frequency of dietary habits^**a**^	Willingness^**b**^	OR	OR (95% CI)	***P***-value
**Smoking cigarettes/day**				
> 20 cigarettes/day	Increase	2.048	0.869–4.831	0.101
	Decrease	1.547	0.717–3.342	0.266
	Quit smoking	3.054	1.277–7.308	0.012
	Uncertain	1.031	0.423–2.516	0.946
11–20 cigarettes/day	Increase	2.067	1.359–3.143	0.001
	Decrease	1.659	1.152–2.389	0.007
	Quit smoking	1.885	1.263–2.813	0.002
	Uncertain	1..740	0.967–3.131	0.064
6–10 cigarettes/day	Increase	1.799	1.392–2.324	< 0.001
	Decrease	1.375	1.081–1.749	0.010
	Quit smoking	1.199	0.903–1.592	0.210
	Uncertain	0.817	0.466–1.433	0.481
1–5 cigarettes/day	Increase	1.645	1.340–2.019	< 0.001
	Decrease	1.191	0.983–1.443	0.074
	Quit smoking	1.097	0.862–1.395	0.452
	Uncertain	0.757	0.478–1.201	0.237
No smoking	Increase	2.126	1.712–2.639	< 0.001
	Decrease	1.349	1.154–1.577	< 0.001
	Quit smoking	1.370	1.206–1.557	< 0.001
	Uncertain	0.825	0.642–1.061	0.134
Total	Increase	2.414	2.212–2.635	< 0.001
	Decrease	1.735	1.617–1.861	< 0.001
	Quit smoking	2.239	1.825–2.747	< 0.001
	Uncertain	1.006	0.842–1.202	0.950
**Alcohol consumption/week**				
Every day	Increase	1.422	1.085–1.865	0.011
	Decrease	1.068	0.831–1.372	0.609
	Quit drinking	1.324	0.855–1.981	0.172
	Uncertain	1.022	0.502–2.080	0.953
4–6 days/week	Increase	1.206	0.972–1.495	0.089
	Decrease	0.975	0.788–1.206	0.815
	Quit drinking	1.228	0.920–1.639	0.163
	Uncertain	0.313	0.163–0.599	< 0.001
2–3 days/week	Increase	1.814	1.460–2.255	< 0.001
	Decrease	1.448	1.196–1.754	< 0.001
	Quit drinking	1.448	1.146–1.831	0.002
	Uncertain	0.788	0.485–1.281	0.337
≤ 1 day/week	Increase	1.723	1.330–2.230	< 0.001
	Decrease	1.379	1.166–1.632	< 0.001
	Quit drinking	1.447	1.175–1.781	< 0.001
	Uncertain	1.048	0.754–1.458	0.779
No drinking	Increase	1.389	0.877–2.200	0.161
	Decrease	1.327	1.078–1.632	0.008
	Quit drinking	1.321	1.140–1.530	< 0.001
	Uncertain	0.987	0.808–1.206	0.898
Total	Increase	2.320	2.126–2.530	< 0.001
	Decrease	1.720	1.608–1.840	< 0.001
	Quit drinking	1.536	1.406–1.679	< 0.001
	Uncertain	0.900	0.775–1.045	0.166
**Sleep duration/day (including lunch break)**				
< 5 h/day	Increase	1.098	0.874–1.379	0.423
	Decrease	1.134	0.819–1.569	0.449
	Uncertain	0.549	0.320–0.942	0.030
5–6 h/day	Increase	1.442	1.185–1.754	< 0.001
	Decrease	1.634	1.301–2.053	< 0.001
	Uncertain	0.858	0.589–1.249	0.424
6–7 h/day	Increase	1.596	1.411–1.806	< 0.001
	Decrease	2.149	1.856–2.488	< 0.001
	Uncertain	1.193	0.925–1.539	0.174
7–8 h/day	Increase	1.334	1.197–1.486	< 0.001
	Decrease	1.798	1.548–2.089	< 0.001
	Uncertain	1.192	0.965–1.472	0.103
> 8 h/day	Increase	2.065	1.689–2.525	< 0.001
	Decrease	1.279	1.016–1.610	0.036
	Uncertain	1.282	0.969–1.697	0.082
Total	Increase	1.491	1.401–1.587	< 0.001
	Decrease	1.736	1.603–1.880	< 0.001
	Uncertain	1.057	0.932–1.199	0.388
**Physical exercise time/week**				
< 30 min/week	Increase	0.759	0.667–0.863	< 0.001
	Decrease	1.379	1.041–1.828	0.025
	Uncertain	0.447	0.311–0.641	< 0.001
30 min–1 h/week	Increase	1.279	1.153–1.419	< 0.001
	Decrease	1.495	1.265–1.766	< 0.001
	Uncertain	0.753	0.543–1.044	0.088
1–2 h/week	Increase	1.397	1.210–1.613	< 0.001
	Decrease	1.641	1.381–1.950	< 0.001
	Uncertain	0.940	0.661–1.336	0.730
2–3 h/week	Increase	1.1–9	0.868–1.417	0.407
	Decrease	1.176	0.900–1.536	0.236
	Uncertain	0.982	0.644–1.496	0.931
> 3 h/week	Increase	1.162	0.862–1.566	0.326
	Decrease	1.123	0.753–1.675	0.569
	Uncertain	1.312	0.819–2.103	0.259
Total	Increase	1.270	1.191–1.117	< 0.001
	Decrease	1.588	1.451–1.739	< 0.001
	Uncertain	0.828	0.705–0.971	0.020

^a^Baseline intake frequency of behavioral habits was regarded as a stratification factor

^b^Unwillingness to change behavior habits was regarded as a reference group

^c^Frontline epidemic prevention experience was classified into dichotomous variables in the models (regarding “have the frontline epidemic prevention experience” as 1, and “no frontline epidemic prevention experience” as 2), adjusting for other characteristic factors (gender, age, BMI, education level marital status, occupation, and main living place)

## Discussion

COVID-19 has quickly spread worldwide since the outbreak in Wuhan. Zhou et al. [27] reported that health care workers are more susceptible to COVID-19. Many frontline epidemic prevention workers have been infected by coronavirus at work in almost in every country, and some of them have died in the fight against the epidemic [28]. Frontline epidemic prevention workers are the most valuable resource of every country, and the Lancet has suggested the government protect health care workers [12].

Frontline epidemic prevention workers are suffering from heavy workloads, high risk of infection, discrimination from other citizens, and so on. Most of them have experienced mental pressure, depression, and anxiety during the outbreak of COVID-19, especially those who have worked in seriously affected areas [10, 13, 29, 30]. Chew et al. [16, 17] found that regardless of the volume of cases or deaths, health care workers from all five Asian-Pacific countries were vulnerable to psychological distress during the outbreak of COVID-19, and Tan et al. [18] reported that the prevalence of psychological adversity was higher among nonmedical health care workers than among health care workers during the COVID-19 outbreak in Singapore. Chew et al. [17] also found that psychological disorders were significantly associated with physical symptoms. Le et al. and Le et al. [29, 30] reported that depression and anxiety were associated with being in contact with potential COVID-19 patients. Therefore, psychological interventions are urgent for protecting frontline epidemic prevention workers from diverse psychological outcomes during COVID-19.

In addition to a negative effect on psychology, COVID-19 also has a negative impact on dietary and behavioral habits. The pandemic has enforced numerous restrictions on daily living including social distancing, isolation, and home confinement [4]. These measures may have caused many negative changes in lifestyle behaviors, including eating more unhealthy diets, engaging in less physical activity, smoking more cigarettes and consuming more alcohol, and increases in depression and anxiety, which makes individuals more susceptible to chronic diseases as well as COVID-19 [19, 29–31]. Husain et al. reported that COVID-19 changed dietary habits and lifestyle behaviors in Kuwait, including skipping breakfast, having more late-night snacks and daytime naps, and getting less physical activity and nighttime sleep [32]. Our previous study showed that high epidemic concern was positively correlated with poor dietary habits during the COVID-19 outbreak [33]. Our current study focused on frontline epidemic prevention workers who had frontline epidemic prevention experience during the epidemic outbreak in China. We aimed to identify their baseline dietary and behavioral habits before the COVID-19 period and further explore their willingness to change dietary and behavioral habits after experiencing the outbreak of COVID-19 based on a cross-sectional study. To further explore targeted strategies, the susceptible subgroups were identified by stratified analyses for subsequent protection from being infected with COVID-19.

The average age of 9402 frontline epidemic prevention workers in this study was 28.09 ± 7.50 years old, which was younger than that of health care workers in five Asian-Pacific countries [16]. Our results found that almost all dietary and behavioral habits of frontline epidemic prevention workers were worse than those of other participants before the epidemic. Christensen et al. also reported that health care workers had a high prevalence of obesity and musculoskeletal pain and low physical capacities [34]. These conclusions might be ascribed to their poor lifestyles, night shifts, and other characteristics of their occupation.

After experiencing the outbreak of COVID-19, most frontline epidemic prevention workers tended to choose healthy dietary and behavioral habits. The numerical results showed that frontline epidemic prevention experience exerted a huge impact on dietary and behavioral habits, and frontline epidemic prevention workers expressed a greater willingness to adopt healthy dietary and behavioral habits than others. These meaningful changes in lifestyle behaviors during COVID-19 were consistent with what Balanzá-Martínez reported [35]; however, they also found that being an essential worker, having worse self-rated health, obtaining a positive screening for depression/anxiety, and making substantial changes in diet/nutrition and sleep were all associated with poorer lifestyles.

It is worth noting that some frontline epidemic prevention workers with bad dietary and behavioral habits before COVID-19 expressed a willingness to adopt worse habits after experiencing the outbreak of COVID-19. As Di Renzo et al. reported, the epidemic clearly affected the dietary and behavioral habits of the Italian population during COVID-19 [27]. Healthy dietary and behavioral habits could boost immunity, prevent or treat chronic diseases, including hypertension, diabetes, and insomnia [26, 36, 37] and prolong life [38]. People with bad dietary and behavioral habits or chronic diseases have been more susceptible to COVID-19 [21]. Ammar reported that isolation was a necessary measure to protect public health, but the results indicated that isolation altered physical activity and eating behaviors in a health-compromising direction [19]. These results might be ascribed to boredom, anxiety, out of control eating, more snacking between meals, social distancing, and isolation at home during the COVID-19 period [39].

All the above findings suggest that dietary and behavioral intervention policies are urgently needed to protect frontline epidemic prevention workers, especially those that had poor dietary and behavioral habits before the COVID-19 outbreak. Internet-based cognitive behavioral therapy may be a good choice for treating frontline epidemic prevention workers with psychiatric symptoms and addressing unhealthy dietary and behavior lifestyles during the pandemic [40]. Online therapy, rather than face-to-face meetings, has the potential to reduce body dissatisfaction, anxiety, and depression during the COVID-19 period [41–43]. It is necessary to establish and improve the working conditions for those in epidemic prevention work. In addition, receiving medical training is needed before fighting the epidemic. Another suggestion is that frontline epidemic prevention workers should be in good health without prior medical conditions [16].

We acknowledge certain limitations of the study. First, a cross-sectional study does not allow us to assess the causality between frontline epidemic prevention experience and willingness to change dietary and behavioral habits after experiencing the outbreak of COVID-19. Second, questionnaires were conducted without strict control measures. However, our study has several advantages, including a large sample size and a wide range of types of frontline epidemic prevention workers, including medical workers, community workers, policemen, volunteers, and others who took part in the fight against COVID-19. Moreover, our questionnaires were conducted after the outbreak of COVID-19 in China, while most of the previous questionnaires had been conducted during the outbreak of COVID-19. Therefore, our findings are helpful for protecting more frontline epidemic prevention workers after the initial outbreak of COVID-19 by examining the adopting of healthy dietary and behavioral habits.

## Conclusions

In summary, our conclusions were based on a large sample size, anonymity, and a confidential internet questionnaire. Our findings suggest that the dietary and behavioral habits of frontline epidemic prevention workers were worse than those of other participants before the COVID-19 outbreak. The frontline epidemic prevention experience exerted a huge impact on their dietary and behavioral habits, and frontline epidemic prevention workers had the willingness to adopt healthy dietary and behavioral habits after experiencing the outbreak of COVID-19. Some frontline epidemic prevention workers with poor dietary and behavioral habits before the COVID-19 outbreak expressed a willingness to engage in worse habits after experiencing the outbreak of COVID-19. Therefore, dietary and behavioral intervention policies are urgently needed to protect their health, especially for those who have poor dietary and behavioral habits.

## Data Availability

All data in the study can be accessed from the corresponding author upon request.

## Abbreviations

COVID-19: 2019 Novel coronavirus disease
WHO: World health Organization
BMI: Body mass index
